# Peruvian oribatid mites (Acari, Oribatida) from the German Biological Expedition, with description of a new species of the genus *Pergalumna*

**DOI:** 10.3897/zookeys.487.9335

**Published:** 2015-03-16

**Authors:** Sergey G. Ermilov, Dariusz J. Gwiazdowicz

**Affiliations:** 1 Tyumen State University, Tyumen, Russia; 2 Poznan University of Life Sciences, Poznań, Poland

**Keywords:** Oribatid mites, fauna, checklist, new record, new species, *Pergalumna*, Peru

## Abstract

The present study is based on oribatid mite material (Acari, Oribatida) collected during the German Expedition in 2011 in Peru. An annotated checklist of identified oribatid mites, including 16 species from 14 genera and 8 families, is provided. Thirteen species and two genera (*Notohermannia*, *Zetomimus*) are recorded for the first time in Peru; the genus *Notohermannia* and species *Notohermannia obtusa* are recorded for the first time in the Neotropical region. A new species of the genus *Pergalumna* (Galumnidae), *Pergalumna paraboliviana* **sp. n.**, is described. The new species is most similar to *Pergalumna boliviana* Ermilov, 2013 from Bolivia, however, it differs from the latter by the body size, morphology of porose areas *A1* and the presence of interlamellar setae.

## Introduction

Oribatid mites (Acari: Oribatida) of Peru are poorly known (see Hammer 1961; Balogh 1962a, b, 1986; Beck 1962a, b; Balogh and Balogh 1985; Woas 1986; Starý 1992; Wunderle 1992; Niedbała 1982, 2004; Franklin et al. 2006; Schatz 2006).

Our investigation is based on Peruvian material collected during a one-month German Expedition organized by Bavarian State Collection of Zoology in September (second half) and October (first half) 2011. The primary goal of the paper is to present the checklist of the identified species with new records for Peru as well as for the Neotropical region.

In the course of taxonomic identification, we found one new species of the genus *Pergalumna* (Galumnidae). The secondary goal of this paper is to describe and illustrate this species. *Pergalumna* is a large genus that was proposed by Grandjean (1936) with *Oribata nervosa* Berlese, 1914 as type species. Currently, it comprises more than 130 species having a cosmopolitan distribution collectively (Subías 2004, updated 2014). The generic characters of the genus are summarized by Ermilov et al. (2013a), and an identification key to known species from the Neotropical region was presented by Ermilov et al. (2014a).

## Materials and methods

Samples were collected from six localities in Peru, Panguana, basin of the Río Yuyapichis (9°36'49.32"S, 74°56'8.16"W) by D.J. Gwiazdowicz:

- Locality 6, rotting wood, 26.09.2011;
- Locality 12, forest litter, 29.09.2011;
- Locality 16, forest litter, 29.09.2011;
- Locality 28, rotting wood, 3.10.2011;
- Locality 29, forest litter, 5.10.2011;
- Locality 44, forest litter, 8.10.2011.

Specimens were mounted in lactic acid on temporary cavity slides for measurement and illustration. The body length was measured in lateral view, from the tip of the rostrum to the posterior edge of the ventral plate. The notogastral width refers to the maximum width in dorsal aspect. Lengths of body setae were measured in lateral aspect. All body measurements are presented in micrometers. Formulas for leg setation are given in parentheses according to the sequence trochanter–femur–genu–tibia–tarsus (famulus included). Formulas for leg solenidia are given in square brackets according to the sequence genu–tibia–tarsus. Microscope figures were made with a drawing tube using a Carl Zeiss transmission light microscope “Axioskop-2 Plus”. General terminology used in this paper follows that of Grandjean (summarized by Norton and Behan-Pelletier 2009).

## Checklist^1^

This annotated checklist includes the specific localities where oribatid mites were collected, and notes new records and overall known distribution^2^.

**Hypochthoniidae**

– *Eohypochthonius becki* Balogh & Mahunka, 1979. Locality: 29 (7 spec.). Distribution: Neotropical region. First record for Peru.

**Nanhermanniidae**

– *Cyrthermannia guadeloupensis* Mahunka, 1985. Locality: 29 (1 spec.). Distribution: Neotropical region. First record for Peru.

– *Notohermannia obtusa* Balogh, 1985. Locality: 29 (10 spec.). Distribution: Australia. First record of genus for Peru and the Neotropical region.

**Oppiidae**

– *Brachioppia cuscensis* Hammer, 1961. Locality: 29 (1 spec.). Distribution: Neotropical region, India and Japan.

– *Gittella variabilis* Ermilov, Sandmann, Marian & Maraun, 2013. Locality: 44 (2 spec.). Distribution: Ecuador. First record for Peru.

– Ramusella (Insculptoppia) merimna (Balogh & Mahunka, 1977). Locality: 29 (1 spec.). Distribution: Neotropical region. First record for Peru.

**Rhynchoribatidae**

– *Rhynchoribates spathulatus* Balogh & Mahunka, 1969. Locality: 28 (1 spec.). Distribution: Neotropical region. First record for Peru.

**Ceratozetidae**

– *Zetomimus naias* Behan-Pelletier, 1998. Locality: 16 (3 spec.). Distribution: Neotropical region. First record of genus for Peru.

**Haplozetidae**

– *Protoribates paracapucinus* (Mahunka, 1988). Localities: 12 (5 spec.), 16 (19 spec.). Distribution: Ethiopian, Neotropical, Oriental and Palaearctic regions. First record for Peru.

– Trachyoribates (Rostrozetes) ovulum Berlese, 1908 sensu Beck (1965) as *Rostrozetes foveolatus* Sellnick, 1925. Localities: 16 (3 spec.), 29 (24 spec.). Distribution: Cosmopolitan.

**Scheloribatidae**

– Scheloribates (Scheloribates) praeincisus acuticlava (Pérez-Íñigo & Baggio, 1986). Locality: 28 (1 spec.). Distribution: Neotropical region. First record for Peru.

**Galumnidae**

– *Carinogalumna clericata* (Berlese, 1914). Locality: 28 (1 spec.). Distribution: Neotropical region. First record for Peru.

– Galumna (Galumna) cf. flabellifera Hammer, 1958. Localities: 6 (1 spec.), 16 (44 spec.), 29 (229 spec.), 44 (4 spec.). Distribution: pantropics and subtropics. First record for Peru.

– *Pergalumna bifissurata* Hammer, 1972. Localities: 28 (2 spec.), 29 (1 spec.). Distribution: Polynesia and Neotropical region. First record for Peru.

– *Pergalumna decoratissima* Pérez-Íñigo & Baggio, 1986. Locality: 44 (4 spec.). Distribution: Neotropical region. First record for Peru.

– *Pergalumna paraboliviana* sp. n. Localities: 28 (3 spec.), 44 (2 spec.). Distribution: Peru.

In the course of the taxonomic studies of materials, we identified 16 species belonging to 14 genera and 8 families. Of these, 13 species and two genera, *Notohermannia* Balogh, 1985 and *Zetomimus* Hull, 1916, are recorded for the first time in Peru; genus *Notohermannia* and species *Notohermannia obtusa* are recorded for the first time in the Neotropical region.

## Taxonomy

### Pergalumna paraboliviana sp. n.

Taxon classificationAnimaliaOribatidaGalumnidae

http://zoobank.org/EEBE0D21-510C-470E-B1E6-73D18F387BEC

Figs 1 , 2 , 3–5

#### Diagnosis.

Body size: 531–697 × 365–448. Body surface punctate and with striate bands. Rostrum pointed. Rostral, lamellar and interlamellar setae well developed, barbed. Bothridial setae setiform, ciliate unilaterally. Lamellar and sublamellar lines parallel, curving backwards. Anterior notogastral margin not present. Notogaster with three pairs of porose areas: *Aa* and *A3* oval, *A1* slightly triangular, longitudinally elongated. Median pore absent. Adanal setae *ad*_3_ inserted laterally or antero-laterally to lyrifissures *iad*. Postanal porose area oval.

#### Description.

*Measurements*. Body length: 614 (holotype, male), 531–614 (three paratypes: all males) to 697 (paratype: one female); notogaster width: 365 (holotype), 398 (three paratypes: all males) to 448 (paratype: one female).

*Integument*. Body color light brown to brown. Body surface punctate. Ventral part of pteromorphs with slightly developed reticulate pattern in one paratype. Prodorsum with one transverse and two longitudinal striate bands (*s*): transverse band located anterior to insertions of interlamellar setae; longitudinal bands parallel, each located from the transverse band medially to insertions of lamellar setae. Posterior part of notogaster with two parallel, longitudinal striate bands located medially to notogastral alveoli *h*_1_. Between these longitudinal bands, two arcuate bands present, which fused medially by the transverse band. Ventral body side with one pair of diagonal striate bands nearly of pedotecta I (Pd I), one transverse striate band located anteriorly to genital plates, two lateral, transversal striate bands located between genital and anal plates, and one arcuate striate band located posteriorly to anal plates, extending marginally into the ano-adanal region. All striate bands well visible only in light colored or dissected specimens.

*Prodorsum*. Rostrum pointed (see in dorso-lateral and frontal views). Rostral (*ro*, 57–61 in males to 82 in female), lamellar (*le*, 49–57 in males to 69 in female) and interlamellar (*in*, 110–118 in males to 127 in female) setae simple, barbed; lamellar setae thinnest, interlamellar setae thickest. Bothridial setae (*ss*, 159–172 in males to 205 in female) setiform, densely ciliate unilaterally. Exobothridial setae absent. Lamellar and sublamellar lines distinct, parallel, curving backwards. Insertions of lamellar setae distanced from lamellar lines. Porose areas *Ad* absent.

*Notogaster*. Anterior notogastral margin not developed. Dorsophragmata of medium size, longitudinally elongated. Notogastral setae represented by 10 pairs of alveoli. Three pairs of porose areas with distinct margins: *Aa* (20–24 × 14–16) and *A3* (12–16 × 8–12) oval, *A1* (36–45 × 8–20) slightly triangular, longitudinally elongated. Porose areas *Aa* located between notogastral alveoli *la* and *lm*. Median pore absent. All lyrifissures distinct; *im* located latero-anteriorly to *A1*. Opisthonotal gland openings (*gla*) located laterally to *A1*.

*Gnathosoma*. Morphology of subcapitulum, palps and chelicerae typical for *Pergalumna* (see Engelbrecht 1972; Ermilov et al. 2011, 2014b, c). Subcapitulum longer than wide (127–131 × 114–118). Subcapitular setae simple, slightly barbed; *a* (32) longer than *m* (16–20) and *h* (20). Two pairs of adoral setae (*or*_1_, *or*_2_, 12) setiform, hook-like distally, barbed. Palps (77–82) with setation 0–2–1–3–9(+ω). Solenidion attached to eupathidium, both located on dorsal tubercle. Chelicerae (143–147 to 196 in female) with two setiform, barbed setae; *cha* (65–69) longer than *chb* (45–49). Trägårdh’s organ distinct.

*Epimeral and lateral podosomal regions*. Apodemes 1, 2, sejugal and 3 well visible. Four pairs of setiform, smooth epimeral setae observed; setal formula: 1–0–1–2. Setae *3b* (12) longer than *1a*, *4a* and *4b* (6–8). Pedotecta II (Pd II) scale-like in lateral view, slightly triangular, rounded distally in ventral view. Discidia (*dis*) narrowly triangular. Circumpedal carinae (*cp*) distinct, directed to setae *3b*.

*Anogenital region*. Six pairs of genital (*g*_1_, *g*_2_, 8–10; *g*_3_–*g*_6_, 6–8), one pair of aggenital (*ag*, 6–8), two pairs of anal (*an*_1_, *an*_2_, 10–12) and three pairs of adanal (*ad*_1_–*ad*_3_, 12–16) setae thin, smooth. Anterior parts of genital plates with two setae. Adanal setae *ad*_3_ inserted laterally or antero-laterally to lyrifissures *iad.* Postanal porose area oval (16–20 × 6–10).

*Legs*. Morphology of leg segments, setae and solenidia typical for *Pergalumna* (see Engelbrecht 1972; Ermilov et al. 2010, 2011, 2014c). Formulas of leg setation and solenidia: I (1–4–3–4–20) [1–2–2], II (1–4–3–4–15) [1–1–2], III (1–2–1–3–15) [1–1–0], IV (1–2–2–3–12) [0–1–0]; homology of setae and solenidia indicated in Table 1. Solenidion φ on tibiae IV inserted in the middle of dorsal parts.

**Figure 1.**
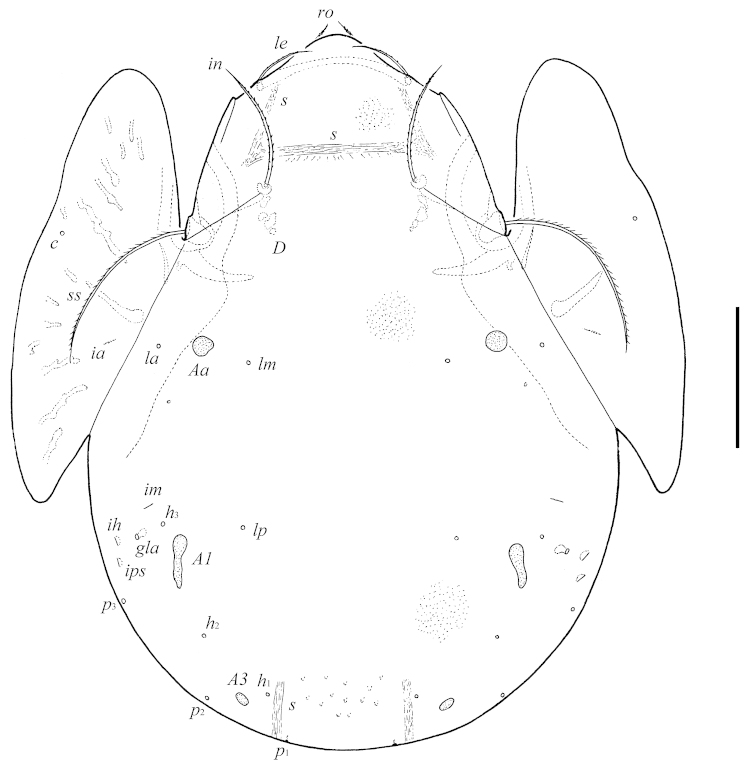
*Pergalumna paraboliviana* sp. n.: dorsal view. Scale bar 100 μm.

**Figure 2.**
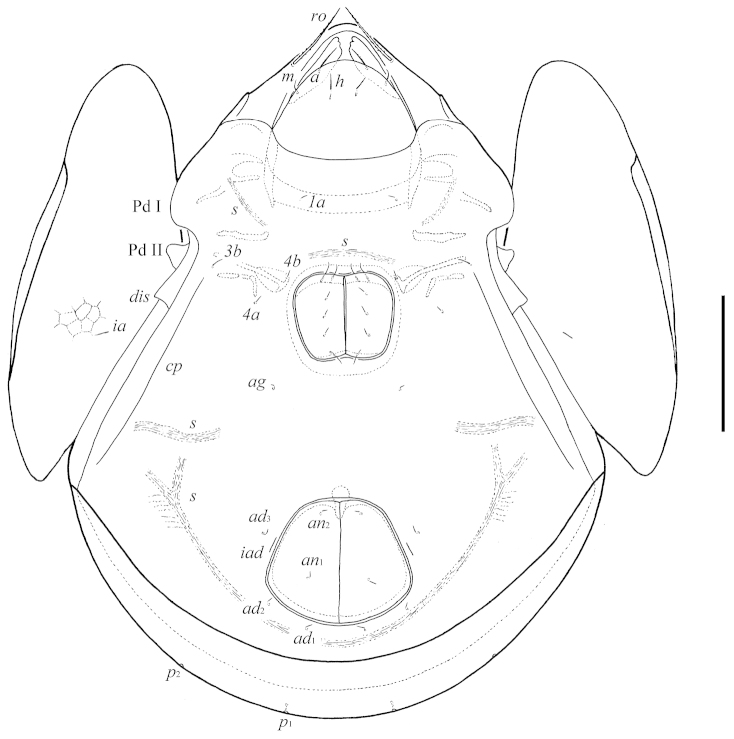
*Pergalumna paraboliviana* sp. n.: ventral view (legs not illustrated). Scale bar 100 μm.

**Figures 3–5.**
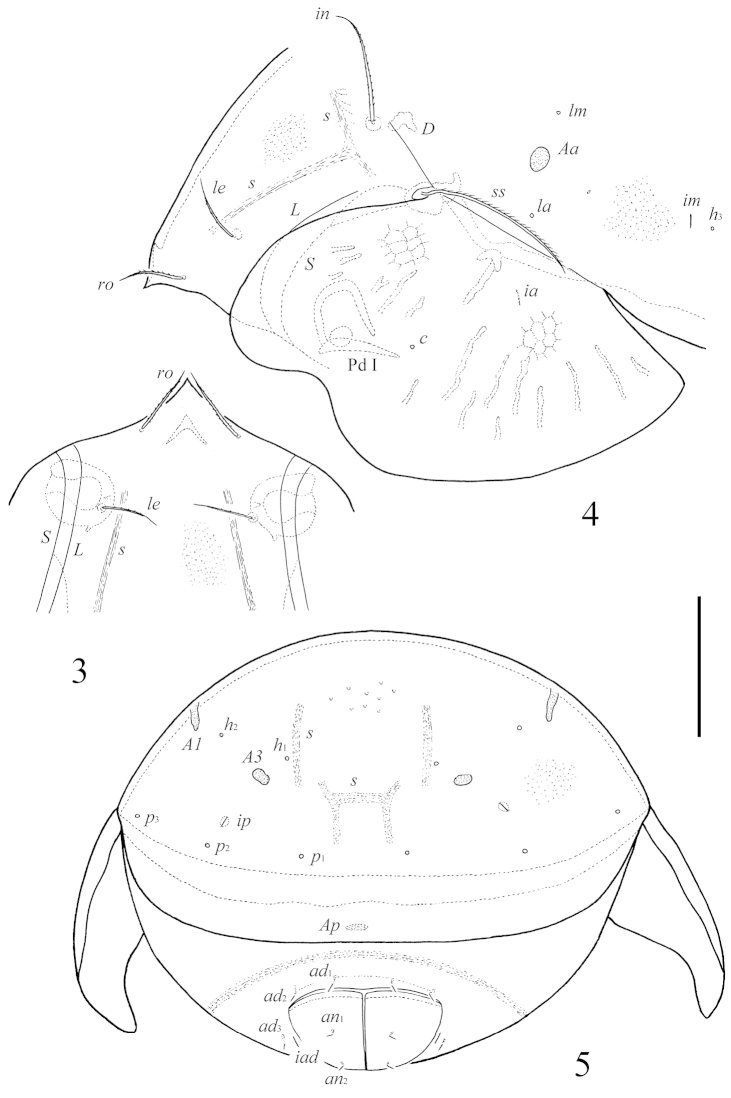
*Pergalumna paraboliviana* sp. n.: **3** rostrum, dorso-frontal view **4** dorso-lateral view of prodorsum, pteromorph and anterior part of notogaster (gnathosoma and legs not illustrated) **5** posterior view. Scale bar 100 μm.

**Table 1.** Leg setation and solenidia of *Pergalumna paraboliviana* sp. n.

Leg	Trochanter	Femur	Genu	Tibia	Tarsus
I	*v*’	*d*, *(l)*, *bv*’’	*(l)*, *v*’, σ	*(l)*, *(v)*, φ_1_, φ_2_	*(ft)*, *(tc)*, *(it)*, *(p)*, *(u)*, *(a)*, *s*, *(pv)*, *v*’, *(pl)*, *l*’’, ɛ, ω_1_, ω_2_
II	*v*’	*d*, *(l)*, *bv*’’	*(l)*, *v*’, σ	*(l)*, *(v)*, φ	*(ft)*, *(tc)*, *(it)*, *(p)*, *(u)*, *(a)*, *s*, *(pv)*, ω_1_, ω_2_
III	*v*’	*d*, *ev*’	*l*’, σ	*l*’, *(v)*, φ	*(ft)*, *(tc)*, *(it)*, *(p)*, *(u)*, *(a)*, *s*, *(pv)*
IV	*v*’	*d*, *ev*’	*d*, *l*’	*l*’, *(v)*, φ	*ft*’’, *(tc)*, *(p)*, *(u)*, *(a)*, *s*, *(pv)*

Roman letters refer to normal setae (ɛ to famulus), Greek letters to solenidia. Single prime (‘) marks setae on anterior and double prime (“) setae on posterior side of the given leg segment. Parentheses refer to a pair of setae.

#### Type deposition.

The holotype is deposited in the collection of the Senckenberg Institution Frankfurt, Germany; three paratypes are deposited in the collection of the Tyumen State University Museum of Zoology, Tyumen, Russia; one pratype is deposited in the collection of the Natural History Museum, Lima, Peru.

#### Etymology.

The prefix *para* is Latin meaning “near” and refers to similarity between the new species and *Pergalumna boliviana* Ermilov, 2013.

#### Remarks.

In having the setiform bothridial setae, pointed rostrum, indeveloped anterior notogastral margin, three pairs of porose areas and striate bands on body, *Pergalumna paraboliviana* sp. n. is most similar to *Pergalumna boliviana* Ermilov, 2013 from Bolivia (see Ermilov and Niedbała 2013). However, it differs from the latter by the larger body size (531–697 × 365–448 versus 415–464 × 282–332 in *Pergalumna boliviana*), elongated, slightly triangular notogastral porose areas *A1* (versus rounded in *Pergalumna boliviana*) and the presence of interlamellar setae (versus represented by alveoli in *Pergalumna boliviana*).

## Supplementary Material

XML Treatment for Pergalumna paraboliviana

## Footnotes

1 Ptyctimous mites are not included in the checklist.

2 See Subías (2004, updated 2014).
